# Lipid‐like Peptides can Stabilize Integral Membrane Proteins for Biophysical and Structural Studies

**DOI:** 10.1002/cbic.201700235

**Published:** 2017-07-17

**Authors:** Katharina Veith, Maria Martinez Molledo, Yasser Almeida Hernandez, Inokentijs Josts, Julius Nitsche, Christian Löw, Henning Tidow

**Affiliations:** ^1^ The Hamburg Centre for Ultrafast Imaging Department of Chemistry Institute for Biochemistry and Molecular Biology University of Hamburg Martin-Luther-King-Platz 6 20146 Hamburg Germany; ^2^ Centre for Structural Systems Biology (CSSB) DESY and European Molecular Biology Laboratory Hamburg Notkestrasse 85 22607 Hamburg Germany; ^3^ Department of Medical Biochemistry and Biophysics Karolinska Institutet Scheeles väg 2 17177 Stockholm Sweden

**Keywords:** membrane proteins, peptergents, peptides, protein stability, X-ray crystallography

## Abstract

A crucial bottleneck in membrane protein structural biology is the difficulty in identifying a detergent that can maintain the stability and functionality of integral membrane proteins (IMPs). Detergents are poor membrane mimics, and their common use in membrane protein crystallography may be one reason for the challenges in obtaining high‐resolution crystal structures of many IMP families. Lipid‐like peptides (LLPs) have detergent‐like properties and have been proposed as alternatives for the solubilization of G protein‐coupled receptors and other membrane proteins. Here, we systematically analyzed the stabilizing effect of LLPs on integral membrane proteins of different families. We found that LLPs could significantly stabilize detergent‐solubilized IMPs in vitro. This stabilizing effect depended on the chemical nature of the LLP and the intrinsic stability of a particular IMP in the detergent. Our results suggest that screening a subset of LLPs is sufficient to stabilize a particular IMP, which can have a substantial impact on the crystallization and quality of the crystal.

## Introduction

Membrane proteins represent about one third of the proteins in living organisms^1^ and play central roles in all physiological processes. Integral membrane proteins (IMPs) are able to shuttle, pump, exchange, bind, and transmit molecules and signals across the membrane on the microsecond to millisecond timescale.^2^

Membrane protein crystallography has made tremendous progress in the last decade(s), and recent advances in cryoelectron microscopy now allows structure determination (to near‐atomic resolution) of large membrane proteins (and complexes thereof) without the need for crystals.^3^ However, the structural biology of integral membrane proteins is still hampered by the instability of many solubilized and purified membrane proteins.^4^ The preparation of a pure, monodisperse, and stable membrane‐protein sample currently seems to be the bottleneck in the structural biology of membrane proteins.^5^ Detergents are commonly used to extract IMPs from their native membrane environment by forming a micelle belt around their hydrophobic transmembrane domains (TMs).^6^, ^7^, ^8^ Although detergents are able to solubilize IMPs, they are poor membrane mimics, and the loss of membrane pressure can induce conformational dynamics, which contribute to increased sample heterogeneity, dramatically reduced thermodynamic stability, and, hence, lower success rates in crystallization.^4^, ^5^, ^8^ For the crystallization of integral membrane proteins, a smaller micelle size achieved by reducing the aliphatic chain length has, in general, a positive effect on the diffraction properties of the crystals owing to easier crystal contact formation. However, shorter chain detergents typically have a higher denaturing potential than their long‐chain counterparts.^9^, ^10^ In addition, the activity of many IMPs in detergents is significantly reduced relative to that in their native lipid environment,^11^, ^12^ which further emphasizes the suboptimal properties of many detergents in membrane protein structural biology.

Lipid‐like peptides—sometimes also called peptergents^13^—have been proposed as alternatives to overcome the detrimental limitations of classical detergents and to expand the toolbox for membrane protein biochemistry.^14^, ^15^, ^16^ These lipid‐like peptides have detergent‐like properties and consist of a short hydrophobic tail produced by repeating copies of nonpolar amino acids and a hydrophilic head group. The head can be neutral or positively (Lys, Arg, His) or negatively (Glu, Asp) charged. Initial biochemical studies with a small set of peptide sequences were highly promising in terms of solubilization efficiencies (if used in addition to detergents) and functional long‐term stability of various extracted and purified IMPs (e.g., glycerol‐3‐phosphate dehydrogenase, photosystem‐I, rhodopsin, and olfactory receptors) compared to classical detergents.^13^, ^15^, ^16^, ^17^, ^18^, ^19^

In this study, we systematically evaluated the effect of lipid‐like peptides on the stability of integral membrane proteins. Thus, we made use of the change in intrinsic fluorescence upon heat unfolding by using a high‐throughput differential scanning fluorimetry device. With this setup, the transition midpoint (*T*_m_) values could be determined with high precision without the need for additional dyes, as used for conventional thermofluor experiments.^20^, ^21^ Lipid‐like peptides (LLPs) of different compositions (amino acids in the hydrophobic tail and the head group) and chain lengths were tested on five different integral membrane proteins ranging from prokaryotic transporters to eukaryotic pumps, and their effects on stability and crystallization were evaluated.

## Results

### Design of lipid‐like peptides

**Figure 1:**
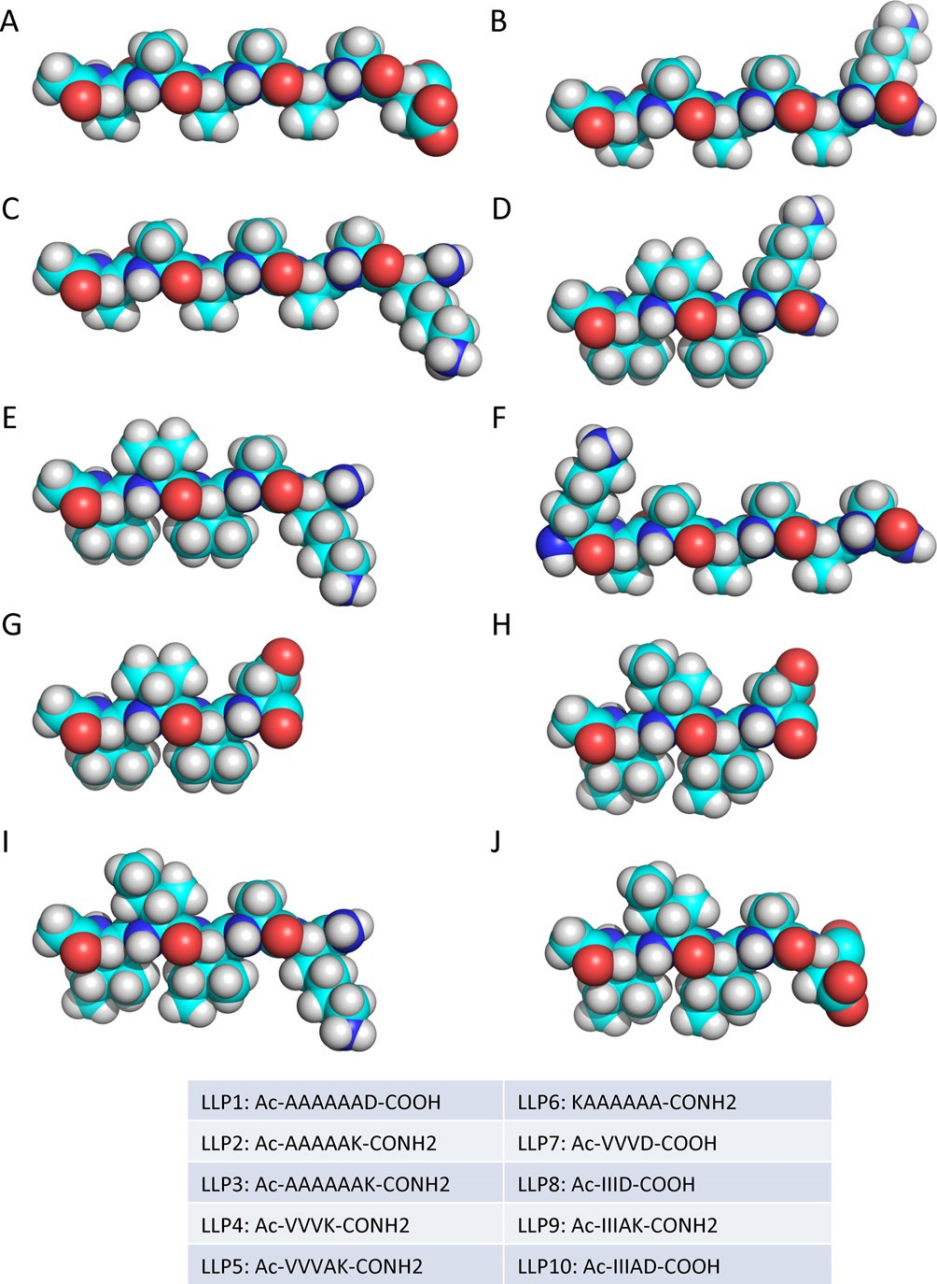
Chemical formulas and ball‐and‐stick models of lipid‐like peptides investigated in this study. A) LLP1, B) LLP2, C) LLP3, D) LLP4, E) LLP5, F) LLP6, G) LLP7, H) LLP8, I) LLP9, J) LLP10. Color code: turquoise: carbon, red: oxygen, blue: nitrogen, and gray: hydrogen.

We designed a series of small, amphiphilic lipid‐like peptides possessing detergent‐like properties and consisting of a short hydrophobic tail and a hydrophilic head group,^18^ and we systematically varied the charges and length of the hydrophobic tail. Lipid‐like peptides are typically between four and seven amino acids long. This corresponds to a length of approximately 2–3 nm, similar to biological phospholipids. The molecular models of all the LLPs used in this study are shown in Figure 1. Most LLPs were acetylated at the N terminus and amidated at the C terminus (see the table in Figure 1). For those LLPs that contained a positively charged amino acid at the N terminus (e.g., LLP6) or a negatively charged amino acid at the C terminus (e.g., LLP1, LLP7, LLP8, and LLP10), the termini were not modified to maintain the charge characteristics (Figure 1).

### Selection of integral membrane proteins as test cases

We chose five different prokaryotic and eukaryotic integral membrane proteins as test cases covering a wide range of sizes, functions, and stabilities. Some of these proteins are very well characterized in terms of structure and function, whereas for others almost no functional or structural data are available. YkoE is a thiamin‐specific vitamin‐transport protein belonging to the energy‐coupling factor (ECF) family of membrane transporters. Its structure was recently determined.^22^ GlpG is an intramembrane protease from *Escherichia coli* belonging to the rhomboid (serine) protease family, and its transmembrane domain is structurally very well characterized.^23^, ^24^, ^25^ ACA8 is a plasma‐membrane Ca^2+^‐ATPase from *Arabidopsis thaliana* belonging to the P‐type ATPase family of ion pumps. ACA8 is autoinhibited in its resting state and becomes activated by binding of Ca^2+^‐calmodulin to its regulatory domain.^26^ ACA8 contains ten transmembrane helices but also three large cytosolic domains. PepT_St_ from *Streptococcus thermophilus* and the hypothetical sugar transporter from *E. coli* both belong to the major facilitator superfamily (MFS) transporter family and are involved in nutrient uptake.^27^, ^28^, ^29^, ^30^, ^31^ Although PepT_St_ has been extensively studied and its structure has been determined in detergent and in a more lipid‐like environment by using the lipidic cubic phase method,^32^, ^33^, ^34^, ^35^ not much is known about the hypothetical sugar transporter from *E. coli*.

### Solubilization of IMPs by using lipid‐like peptides

We first investigated whether the LLPs alone (without additional detergent) could be used to solubilize integral membrane proteins. For this purpose, we used an ACA8–green fluorescent protein (GFP) fusion to follow the solubilization efficiency spectroscopically. We used a systematic approach and tested various LLP concentrations (<0.5 mg mL^−1^) and incubations times (2–24 h), but we could not detect any significant solubilization of ACA8–GFP by the LLPs in the absence of detergents (data not shown). We concluded that although LLPs were previously reported to be able to solubilize GlpD in vitro,^13^ they were not suitable to solubilize IMPs from the lipid bilayer in our hands. This discrepancy can be explained by the fact that GlpD is a monotopic membrane protein (not an IMP) that attaches only with a small fraction of the protein from one side into one leaflet of the lipid bilayer and can therefore be removed much easier from the membrane.^13^ The IMPs used in this study, however, comprise between six and 14 transmembrane helices and are fully integrated in the membrane bilayer. For this reason, they cannot be solubilized with LLPs alone. The previously observed solubilization of G protein‐coupled receptors (GPCRs) during cell‐free protein synthesis (CFPS)^18^ cannot be compared to the setup used in this study, as during CFPS the polypeptide chain is produced in the absence of a lipid bilayer, which can result in the formation of a nonfunctional protein as a result of the lack of tertiary‐structure formation.^36^

### Lipid‐like peptides can stabilize detergent‐solubilized IMPs

Next, we analyzed whether detergent‐solubilized and purified integral membrane proteins could be stabilized against heat denaturation by the LLPs. The thermal stability of a particular IMP is considered to be one of the key parameters that can be used to determine its protein structure successfully.^37^, ^38^ High‐throughput methods to determine the thermal stability of the IMPs were limited in the past and often depended on the use of fluorescent dyes such as 7‐diethylamino‐3‐(4′‐maleimidylphenyl)‐4‐methylcoumarin (CPM) and SYPRO Orange,^21^, ^39^, ^40^ which are not always compatible with the detergents used for IMPs or display different coupling efficiencies depending on the pH. Alternative thermal unfolding methods that can usually not be performed in high‐throughput processes include far‐UV/near‐UV circular dichroism (CD) spectroscopy, which reports on the secondary/tertiary structures,^41^ and differential scanning calorimetry (DSC), which directly measures the absorbed heat upon unfolding but requires a significant amount of protein.^42^

**Figure 2:**
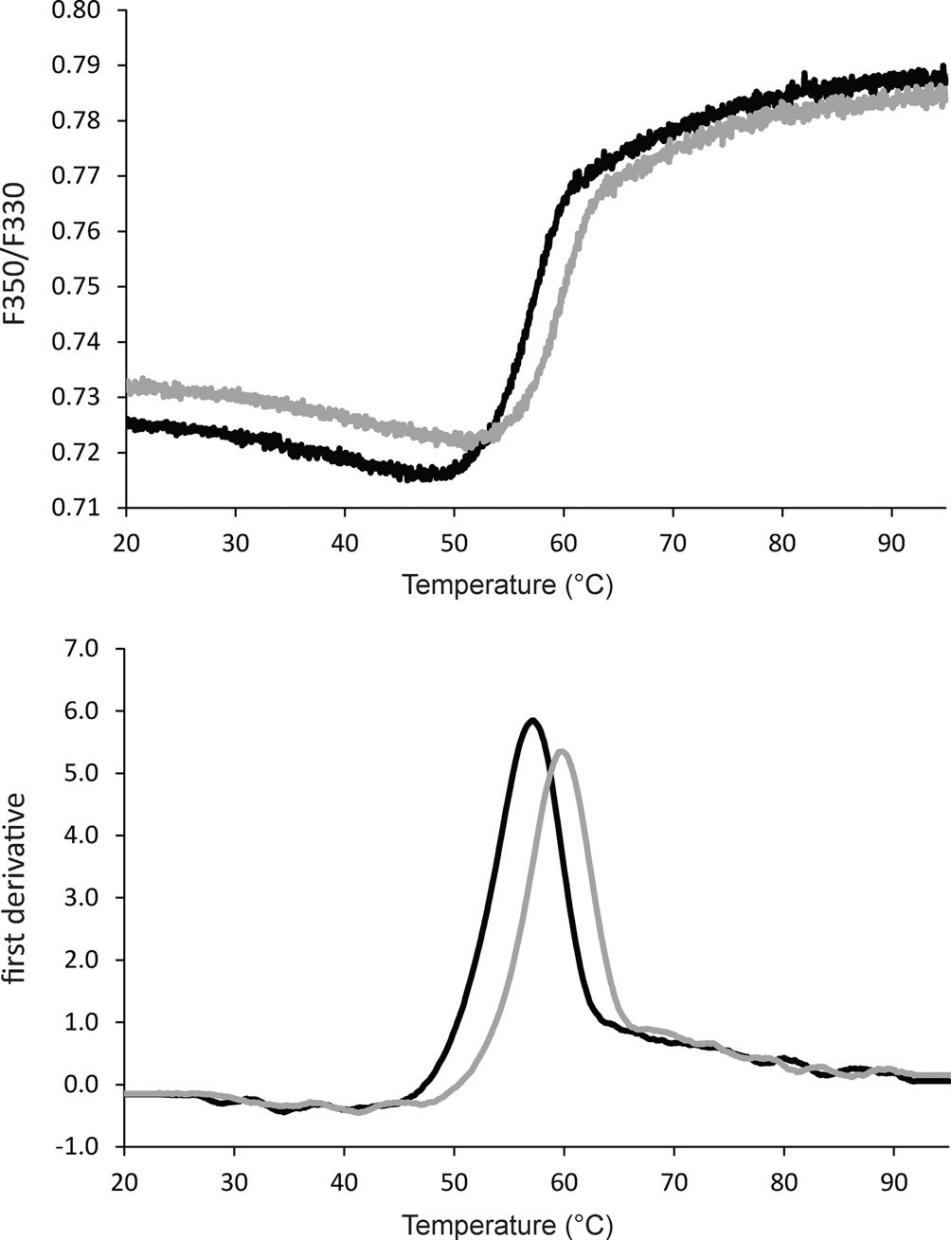
DSF transition curve and first derivative to illustrate the stabilization of IMPs by LLPs. The maximum of the first derivative is the unfolding transition midpoint that was used to quantify the (de)stabilization effect. The data correspond to the hypothetical sugar transporter in the LMNG detergent. Experiments were performed with a protein concentration of 0.7 mg mL^−1^ purified IMP in the absence and presence of 2.5 mm LLP8 at a final DMSO concentration of 4 % by using a heating rate of 1 °C min^−1^.

**Figure 3:**
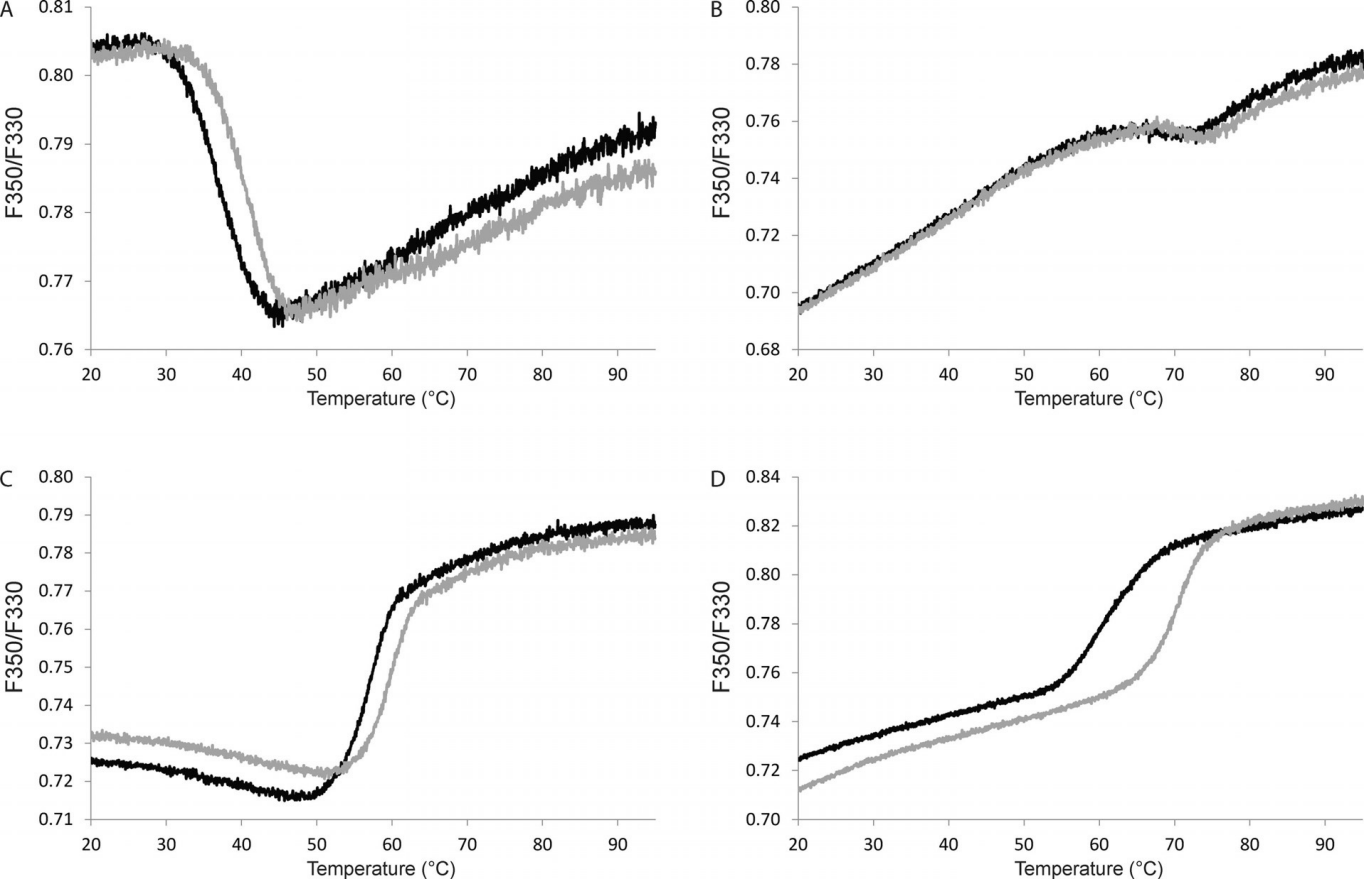
DSF transition curves of various IMP targets in the absence and presence of selected LLPs. Shown are the raw fluorescence data (ratio F350/F330). A) PepT_St_ in NM, B) YkoE in NM, C) hypothetical sugar transporter in LMNG, and D) GlpG in NG. Experiments were performed with IMPs ±2.5 mm LLP (in 4 % DMSO) by using a heating rate of 1 °C min^−1^.

**Figure 4:**
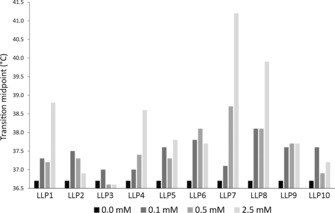
Concentration dependence of the observed transition midpoint. DSF curves were acquired for 1 mg mL^−1^ PepT_St_ in 0.4 % NM in the presence of different concentrations (0–2.5 mm) of various LLPs.

Here, we measured the thermal unfolding of various IMPs in solution by using differential scanning fluorimetry (DSF). We used the intrinsic fluorescence of the aromatic residues and observed the change at *λ*=330 and 350 nm after excitation at *λ*=280 nm (Figure 2). For all of the investigated IMPs, the DSF traces allowed unambiguous assignment of a transition midpoint (*T*_m_) that shifted in response to the addition of the LLPs (Figure 3). For several of the LLPs, this stabilization effect was concentration dependent, whereas for others no significant stabilization and thus no concentration dependence could be observed (Figure 4).

**Figure 5:**
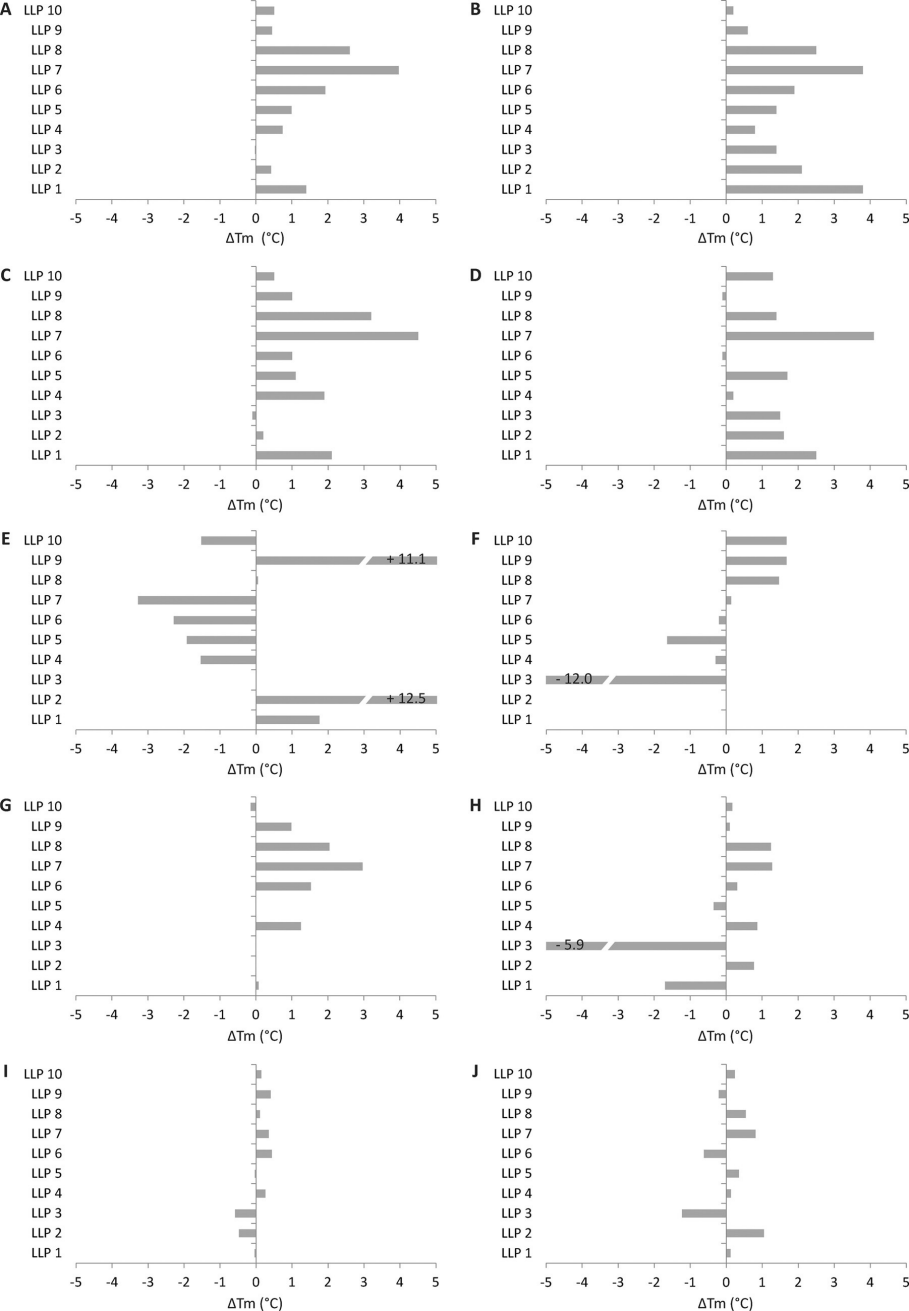
Δ*T*_m_ induced by LLPs. DSF curves of five different integral membrane proteins (in two different detergents each) were acquired in the presence of 2.5 mm of 10 different LLPs. The change in *T*_m_ was calculated from the first derivative. A) Hypothetical sugar transporter (0.7 mg mL^−1^) purified in 0.01 % LMNG, B) hypothetical sugar transporter (0.7 mg mL^−1^) purified in 0.03 % DDM, C) PepT_St_ (1 mg mL^−1^) in 0.4 % NM, D) PepT_St_ (1 mg mL^−1^) in 0.03 % DDM, E) GlpG (0.5 mg mL^−1^) in 0.3 % NG, F) GlpG (0.5 mg mL^−1^) in 0.03 % DDM, G) YkoE (0.5 mg mL^−1^) in 0.4 % NM, H) YkoE (0.5 mg mL^−1^) in 0.2 % DM, I) ACA8 (0.9 mg mL^−1^) in 0.01 % LMNG, and J) ACA8 (0.9 mg mL^−1^) in 0.03 % DDM.

Subsequently, we systematically screened five different IMPs, covering a range from small prokaryotic membrane transporters to large eukaryotic membrane pumps, with regard to their stabilization effect by the addition of the LLPs. Each membrane protein target was purified in two different detergents to be able to compare the effects of detergent/LLP combinations. We found that PepT_St_ from *S. thermophilus* and the hypothetical sugar transporter from *E. coli* were both stabilized by a number of the investigated LLPs, in particular LLP7 and LLP8. The thermal stabilities of these proteins were both increased by up to 4.5 °C. The general stabilizing effect was independent of the detergent in which the IMP had been purified; however, subtle differences exist with respect to the degree of stabilization by the different LLPs. For example, LLP1 stabilized the hypothetical sugar transporter from *E. coli* in dodecylmaltoside (DDM) by 3.8 °C, but the same protein purified in lauryl maltose neopentyl glycol (LMNG) stabilized it only by 1.3 °C (Figure 5 A and B). For YkoE, we also observed a general stabilizing effect of up to 3 °C by many of the LLPs. As notable exceptions, LLP1 and LLP3 destabilized YkoE in decylmaltoside (DM) by 1.8 and 5.9 °C, respectively (Figure 5 G and H). GlpG revealed an interesting stability‐response pattern. GlpG purified in the nonylglucoside (NG) detergent was stabilized by 12.5 and 11.1 °C by the addition of LLP2 and LLP9, respectively (Figure 5 E and F). Several other LLPs had a small destabilizing effect in NG. In the DDM detergent, only LLP8, LLP9, and LLP10 had a stabilizing effect (≈2 °C) on GlpG unfolding (Figure 5 F). Notably, GlpG is highly stable in DDM and nonylmaltoside (NM) with a transition midpoint above 80 °C (data not shown), which probably makes further stabilization by LLPs unlikely. In the NG detergent, the transition midpoint in the absence of the LLPs was 60 °C, which could be increased by >10 °C upon the addition of LLP2 and LLP9. IMPs with lower thermal stability are typically more strongly stabilized by LLPs. However, for ACA8 we observed only minor stabilizing or destabilizing effects (±1 °C) by the addition of the LLPs independent of the detergent used (Figure 5 I and J). This could be due to the fact that this calcium pump is composed of three cytoplasmic domains in addition to the transmembrane domain that accounts for approximately 50 % of the protein, so the addition of the LLPs might have only a negligible effect on the thermal stability.

### Effect of lipid‐like peptides on the crystallization of IMPs

**Figure 6:**
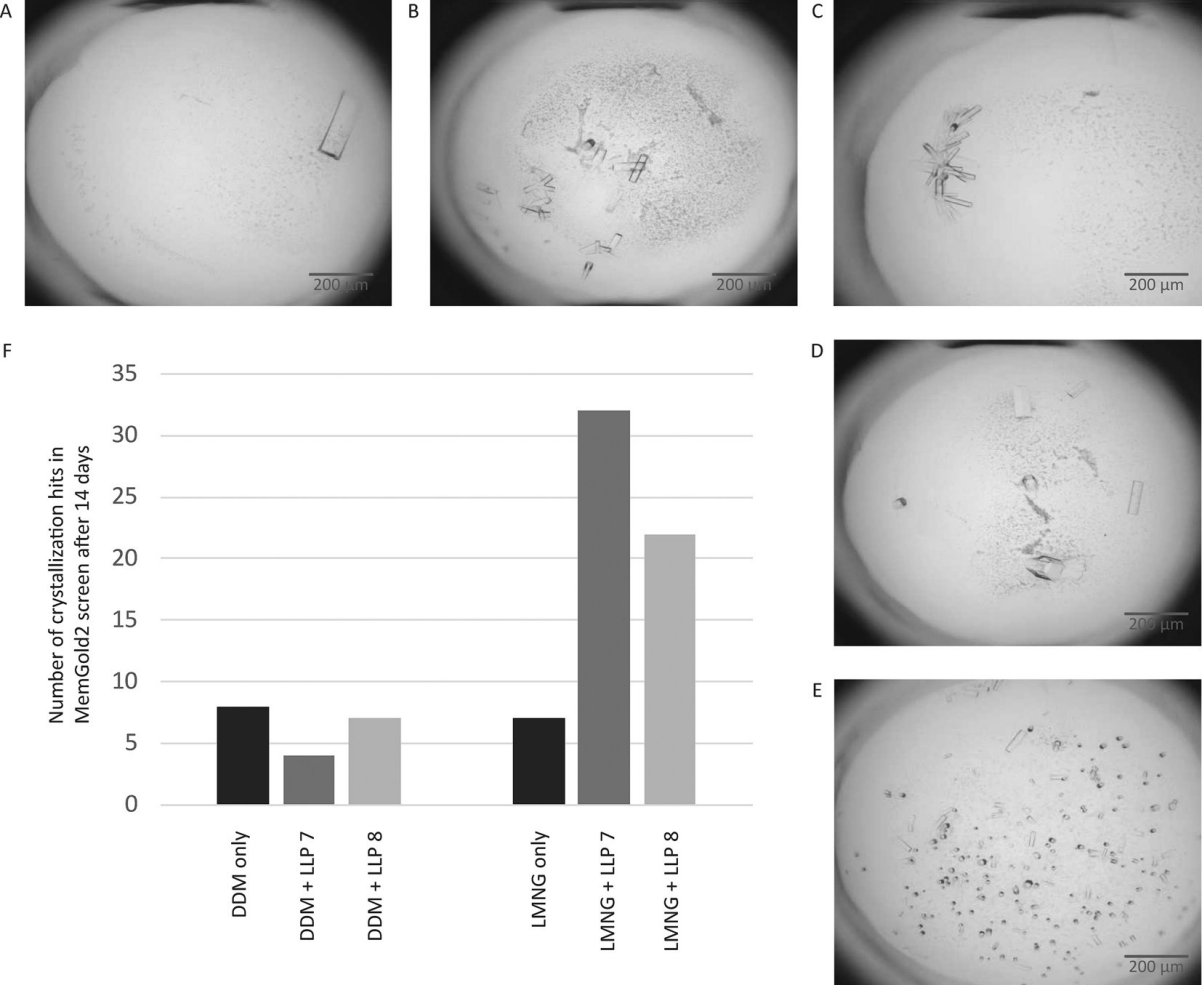
Crystallization statistics. Number of crystallization hits and crystal images of the hypothetical sugar transporter crystallized in the presence of 2.5 mm LLP7 and LLP8. The addition of stabilizing LLP7 and LLP8 to the transporter purified in LMNG led to a significant increase in crystallization hits in the MemGold2 screen.^43^ Representative images of the crystals are shown: A) LLP7, condition E1; B) LLP8, condition D11; C) LLP8, condition E3; D) LLP7, condition B5; and E) LLP8, condition D3. F) Crystallization statistics for the hypothetical sugar transporter and stabilizing LLPs. Protein in gel filtration buffer (20 mm HEPES, pH 7.0, 50 mm NaCl, 5 % glycerol, 0.5 mm TCEP, 0.01 % LMNG, 2.5 mm LLP7/8, 4 % DMSO) was crystallized by mixing it in a 1:1 ratio with precipitant solution (300 nL drop size).

To investigate whether LLPs could have an effect on the crystallization of integral membrane proteins, we set up crystallization trials with the hypothetical sugar transporter from *E. coli* in the presence of the two most‐stabilizing LLPs. By using the commercial MemGold2 crystallization screen,^43^ we observed the formation of large single crystals, suitable for X‐ray diffraction experiments, in 20–30 % of the conditions when using LMNG as the detergent and LLP7 or LLP8 as the additive (Figure 6). Without the presence of the LLPs, crystals only appeared in seven (out of 96) conditions (Figure 6 F). The positive effect of these stabilizing LLPs on the crystallizability of the *E. coli* transporter seemed to depend on the detergent used, as we could not observe this effect with protein purified in DDM. We speculate that the stabilizing effect might be due to increased micelle stiffness (see the Discussion below). These initial crystals co‐crystallized with LLP8 were exposed to X‐rays and were diffracted anisotropically beyond 5 Å resolution in the best direction (Figure S1 in the Supporting Information), which is significantly better than the initial crystal hits obtained for protein crystallized in the absence of LLPs (max. 15 Å diffraction). Therefore, we believe that LLP8 promotes tighter packing of the transporter molecules in the crystal lattice, which results in improved diffraction properties of these crystals. In addition, we could also obtain crystals of GlpG in the presence of stabilizing LLP2 and LLP9 (Figure S2). These crystals appeared under conditions that did not support crystal growth in the absence of the LLPs, which further supports the ability of stabilizing LLPs to enlarge the crystallization space.

## Discussion

The use of detergents with all of their detrimental properties is one of the key limitations in structural studies of IMPs, because they are poor mimics of the lipid bilayer. Numerous detergents are available and have been tested to solubilize, purify, and crystallize IMPs. It is well known that short‐chain detergents such as NM and NG are more denaturing than long‐chain detergents such as DDM^44^ (Figure S3), but at the same time, short‐chain detergents are beneficial for crystallization because of their reduced micelle size. Recent molecular dynamics simulations suggested that this destabilizing effect could be explained by a decrease in α‐helicity in the transmembrane regions (owing to a smaller micelle size of the detergent and, consequently, exposure of a larger fraction of the hydrophobic surface), suboptimal α‐helical packing, and the interpenetration of detergent molecules between TM–helices.^45^ Interestingly, cholesteryl hemisuccinate (CHS) in dodecylmaltoside was found to stabilize selected GPCRs by wedging into hydrophobic crevices and stiffening the detergent micelle as well as by forming a bicelle‐like micelle architecture.^45^, ^46^ The stabilizing effect of certain LLPs on specific IMP target proteins as shown here might be caused by general micelle stabilization (change in micelle size or dynamics by integration of LLPs), specific LLP–IMP interactions, or a combination of both. The fact that various LLPs show opposite effects on various IMPs indicates specific interaction sites on the different proteins. It is tempting to speculate that the extremely pronounced stabilization of GlpG by LLP2 and LLP9 (Figure 5 E and F) might be caused by those peptides acting as substrates. Binding to the active site of GlpG may lead to a tighter interaction of TM–helices and, thus, might confer higher thermal stability.

## Conclusions

In summary, lipid‐like peptides extend the toolbox to stabilize integral membrane proteins in solution not only for structural studies but also for functional studies. Our results indicated that screening a subset of LLPs was sufficient to identify suitable LLPs; this makes them an additional tool for successful structural biology research.

## Experimental Section

**Materials**: The detergents used for purification were from Anatrace (Maumee, OH, USA), and crystallization reagents were from Qiagen. All other chemicals were of analytical grade and were obtained from Roth, unless otherwise stated. Peptides were purchased from GL Biochem (Shanghai, China). Peptide identity was confirmed by LC–MS. LLP stock solutions at 62.5 mm in DMSO were prepared for DSF and crystallization experiments.

**Expression and purification of integral membrane proteins**: The protein PepT_St_ was expressed and purified in the *n*‐dodecyl‐β‐d‐maltopyranoside (DDM) detergent, as described previously.^47^ For the protein batch of PepT_St_ in the *n*‐nonyl‐β‐d‐maltopyranoside (NM) detergent, the protein was initially solubilized and purified in the DM detergent up to the immobilized‐metal affinity chromatography (IMAC) step, after which the detergent was exchanged to NM in the size‐exclusion chromatography (SEC) step.

The hypothetical sugar MFS transporter from *E. coli* (NP_418 146.4) was identified as a suitable target for structural studies by using our recently described pipeline approach.^47^ The gene was cloned into the C‐terminally hexahistidine‐tagged vector pNIC‐CTHF^48^ by using ligation‐independent cloning.^49^ The vectors possessed a tobacco etch virus (TEV) cleavage site for Tag removal. Protein expression in *E. coli* C41, membrane solubilization, and purification by using IMAC and SEC were performed essentially as described,^47^, ^50^, ^51^ except that two different detergents, DDM and LMNG, were used. The protein was eluted from the IMAC column by passing TEV protease over the beads, as the use of high imidazole concentrations for the elution step was found to destabilize the protein strongly.^47^ For the gel‐filtration step, HEPES (20 mm, pH 7.5), NaCl (150 mm), 5 % glycerol, and Tris(2‐carboxyethyl)phosphine (TCEP; 0.5 mm) with the corresponding detergent (0.4 % NM, 0.03 % DDM, 0.01 % LMNG) were used.

YkoE from *Paenibacillus polymyxa* M1 was expressed and purified in a manner similar to that previously described for the homologue from *Bacillus subtilis*.^22^ Briefly, the protein was expressed with an N‐terminal His_6_ tag in *E. coli* Lemo21 strain.^52^ Membranes were isolated and solubilized in 1 % DDM at 4 °C for 1 h. Protein was purified by using Ni‐NTA resin, which was followed by TEV cleavage to remove the His_6_ tag and re‐passage of the digested material over Ni‐NTA resin. The flow through was further purified by using gel‐filtration chromatography in Tris**⋅**HCl (25 mm, pH 7.4), NaCl (300 mm), and 3 % glycerol (using 0.4 % NM or 0.2 % DM).

The transmembrane part of GlpG from *E. coli* (residues 91–270) was prepared as follows: Full‐length GlpG was expressed with an N‐terminal His tag in *E. coli* Lemo21 cells at 20 °C overnight. Membranes were isolated and solubilized with DDM. The protein was purified by using Ni‐NTA resin, and the protein was eluted with 0.3 % NG. Subsequently, GlpG was incubated with chymotrypsin in a ratio of 1:50 at 4 °C for 36 h to remove the N‐terminal soluble domain. The truncated GlpG was purified by gel filtration in Tris**⋅**HCl (25 mm, pH 8.0), NaCl (150 mm), and 0.3 % NG.^23^, ^24^

Full‐length ACA8 from *Arabidopsis thaliana* was expressed in *Saccharomyces cerevisiae* BJ5460^53^ with an N‐terminal His_6_ tag. Membranes were isolated and solubilized in LMNG. The protein was purified by using Ni‐NTA resin in Tris**⋅**HCl (30 mm, pH 8.0), NaCl (300 mm), MgCl_2_ (1 mm), CaCl_2_ (1 mm), β‐mercaptoethanol (2 mm), and 0.01 % LMNG and was eluted with imidazole (150 mm). Fractions containing ACA8 were concentrated and were further purified by size‐exclusion chromatography.

**Thermal stability measurements using nanoDSF**: The stability of the different purified protein preparations in the presence and absence of the LLPs (0–2.5 mm) was followed by using a nanoDSF differential scanning fluorimeter (Prometheus, NanoTemper Technologies, Munich). Here, the intrinsic fluorescence at *λ*=330 and 350 nm after excitation at *λ*=280 nm was used to monitor the fluorescence change upon heat unfolding. Up to 48 samples could be measured in parallel without the addition of a dye. Typically, protein solution (10 μL) at a concentration of 0.5–2 mg mL^−1^ was loaded in a capillary, and the unfolding was then measured at a heating rate of 1 °C min^−1^. The first derivative of the unfolding curves was used to determine the transition midpoint. Given that the LLPs were prepared in DMSO, respective control experiments of the different IMPs in the presence of DMSO were performed. All analyzed samples contained 4 % DMSO. The stability of the analyzed protein was not significantly changed in the presence of 4 % DMSO. For GlpG and ACA8, well‐established activity assays exist,^26^, ^54^, ^55^ which revealed similar activity in 4 % DMSO. In addition, the concentration dependence of the transition midpoint was determined in the range of 0.2 to 5 mg mL^−1^ for each protein batch. Resulting transition midpoints were within 1 °C.

**Crystallization of IMPs in presence of lipid‐like peptides**: Crystallization trials with the commercially available MemGold2 screen^43^ were performed by vapor diffusion in 96‐well sitting‐drop plates at 293 K. The volume of crystallant added to the reservoir was 50 μL, whereas the drops had a total volume of 300 nL and were composed of the *E. coli* transporter sample at a concentration of 5 mg mL^−1^ in the absence and presence of LLPs at 2.5 mm and the crystallant in ratios of 1:2, 1:1, and 2:1. After 14 days of incubation, crystal plates were scored, and the results were compared. The crystals were typically flash frozen in liquid nitrogen without prior soaking in cryobuffer and were tested at the synchrotron beamline P13.^56^

## Conflict of interest

*The authors declare no conflict of interest*.

## Supporting information

As a service to our authors and readers, this journal provides supporting information supplied by the authors. Such materials are peer reviewed and may be re‐organized for online delivery, but are not copy‐edited or typeset. Technical support issues arising from supporting information (other than missing files) should be addressed to the authors.

Supplementary
