# Supplementary material for: Lipid‐like Peptides can Stabilize Integral Membrane Proteins for Biophysical and Structural Studies
Source: Chembiochem. 2017 Jul 17;18(17):1735–42. doi: 10.1002/cbic.201700235 (PMC5601290; doi:10.1002/cbic.201700235)
Supplement: Supplementary file 1 — Supplementary [file CBIC-18-1735-s001.pdf]

CHEMBIOCHEM

Supporting Information

Lipid-like Peptides can Stabilize Integral Membrane Proteins for Biophysical and Structural Studies

Katharina Veith,[a] Maria Martinez Molledo,[b] Yasser Almeida Hernandez,[a] Inokentijs Josts,[a] Julius Nitsche,[a] Christian Löw,*[b, c] and Henning Tidow*[a]

cbic_201700235_sm_miscellaneous_information.pdf

Author Contributions

K.V., M.M.M., Y.A.H., I.J., J.N., C.L., and H.T. performed experiments and analyzed the data. C.L. and H.T. supervised the project. All authors read and approved the manuscript.

Suppl. Fig. S1

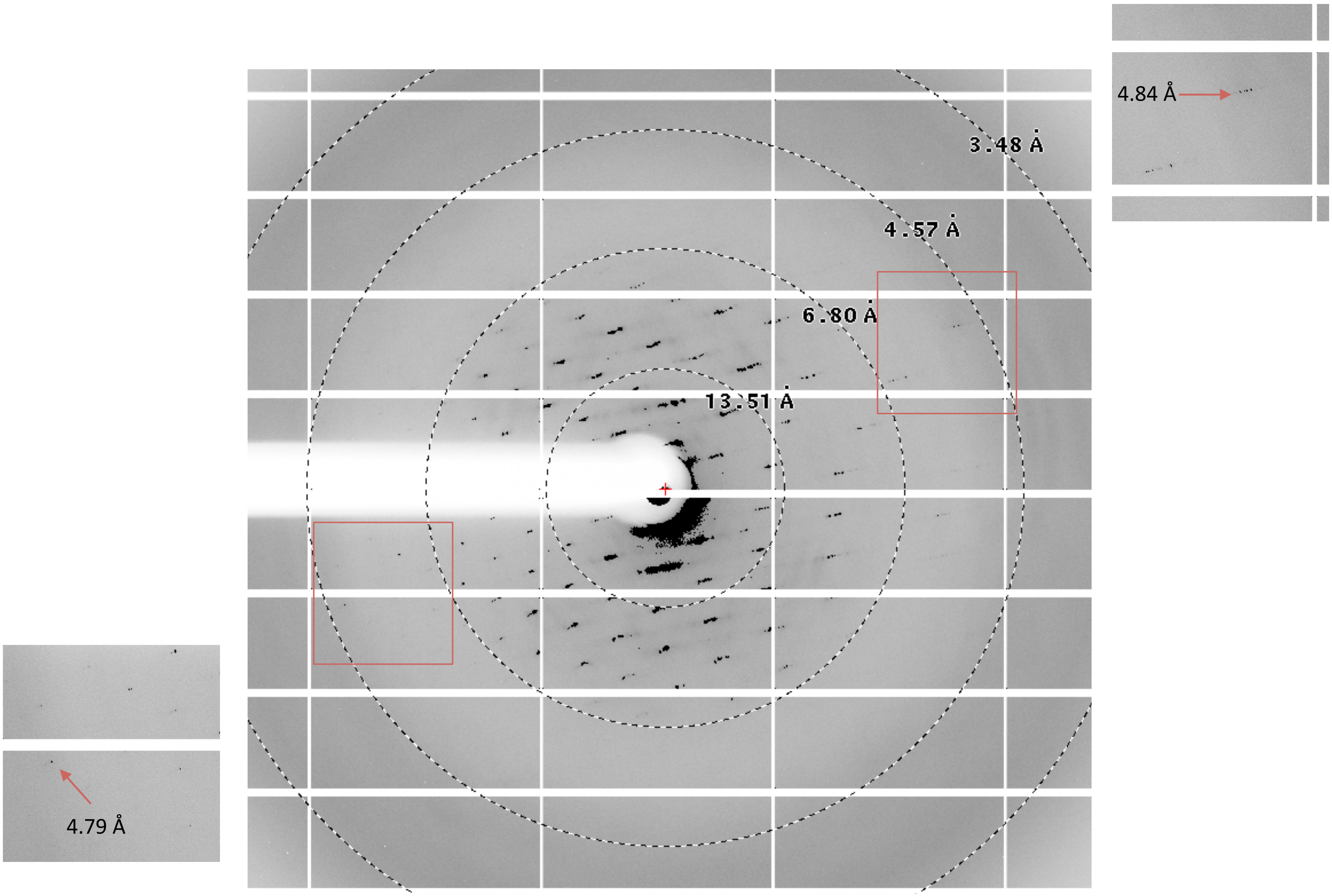

Suppl. Fig. S2

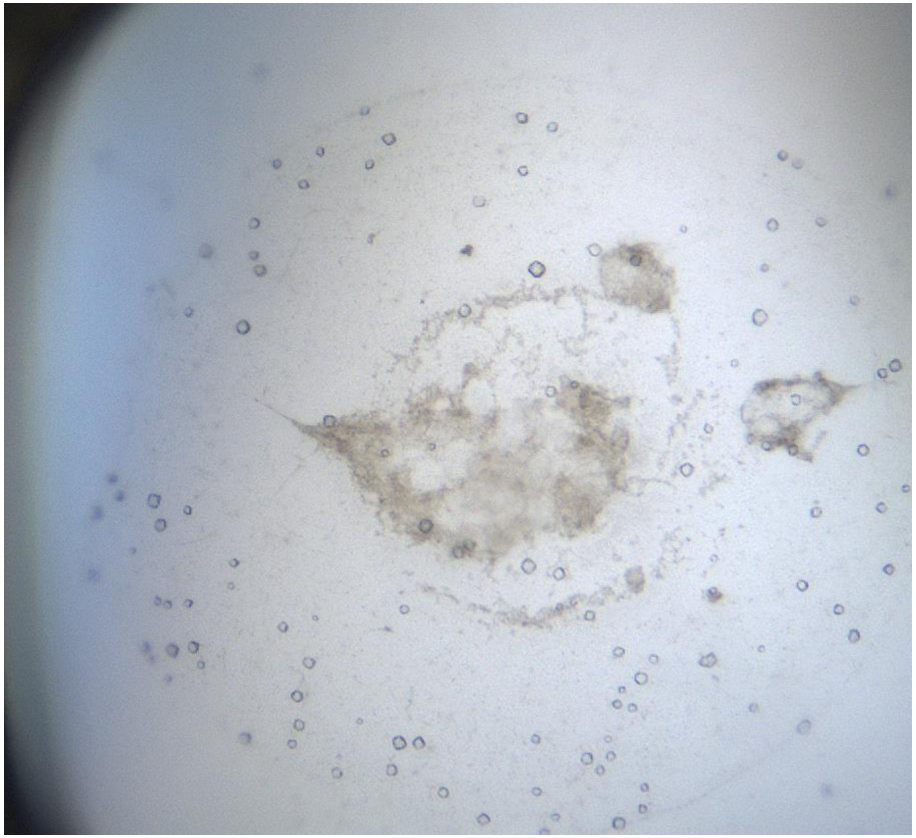

- GlpG 10 mg/ml (purified in 0.3% NG)
- + 2.5 mM LLP2

condition MemGold2_A4:

- 0.04 M Magnesium sulfate heptahydrate
- 0.02 M Sodium chloride
- 0.02 M MES, pH 6.5
- 8 % w/v PEG 1450

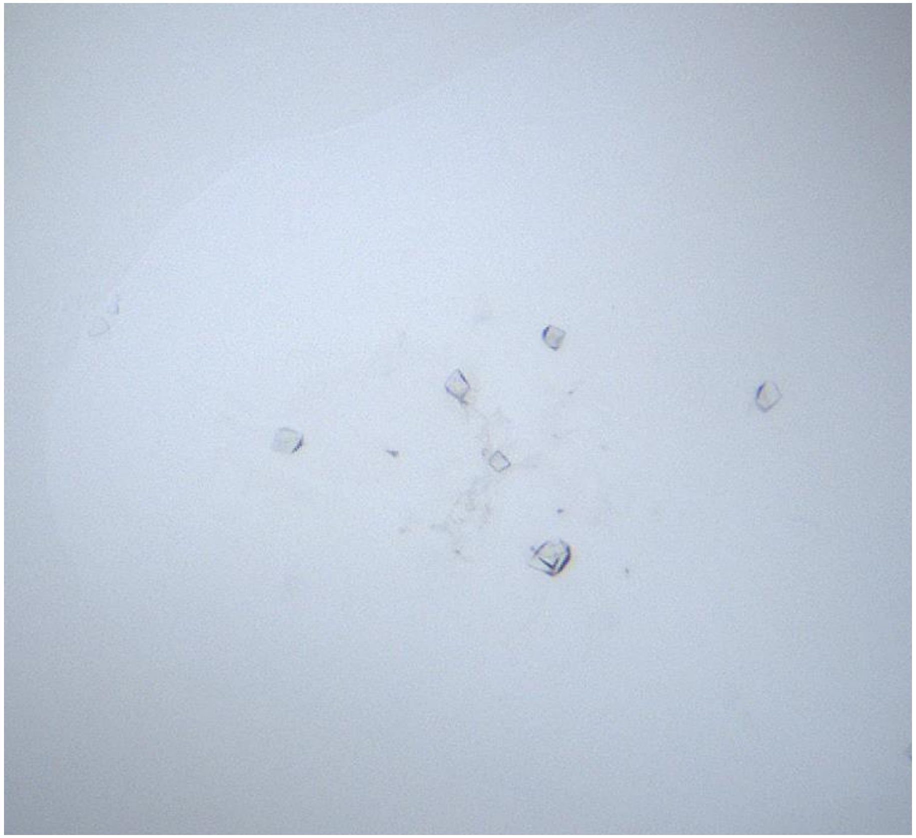

- GlpG 10mg/ml (purified in 0.3% NG)
- + 2.5 mM LLP9

condition MemGold_D8:

- 0.05 M magnesium chloride
- 0.1 M glycine, pH 9.0
- 22 % (v/v) polyethylene glycol 400

Suppl. Fig. S3

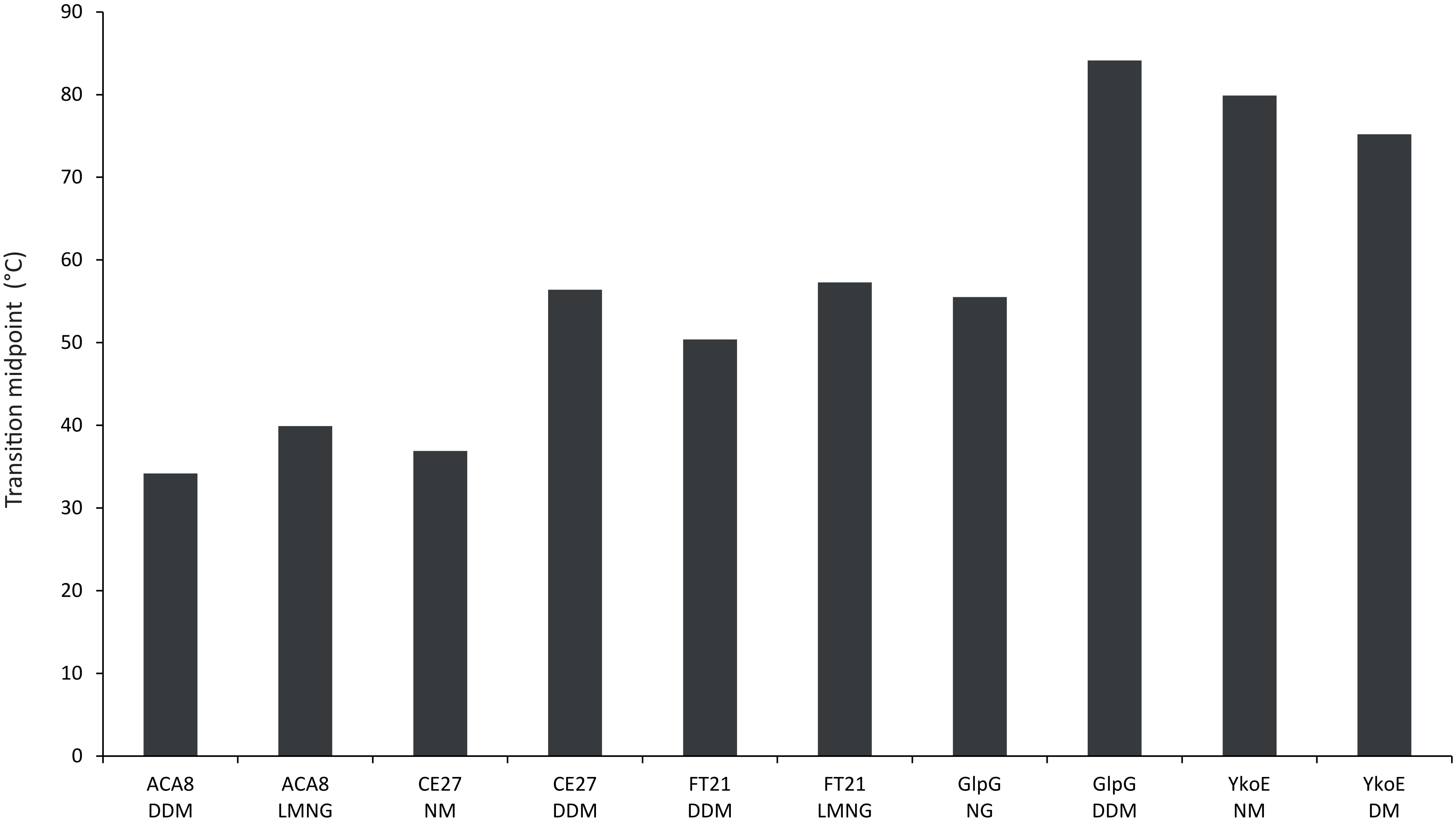

90
80
70
60
50
40
30
20
10
0
Transition midpoint (°C)
ACA8
DDM
ACA8
LMNG
CE27
NM
CE27
DDM
FT21
DDM
FT21
LMNG
GlpG
NG
GlpG
DDM
YkoE
NM
YkoE
DM

Supplementary Figure S1

X-ray diffraction images of the hypothetical sugar transporter from *E. coli* in the presence of LLP8. Diffraction limits are indicated.

Supplementary Figure S2

Crystal images of GlpG (purified in NG) crystallized in the presence of 2.5 mM LLP2 and LLP9. Crystallization conditions are indicated.

Supplementary Figure S3

Absolute T_m of the investigated proteins in the different detergents. The results illustrate an effect of the detergent chain length. HST = hypothetical sugar transporter.
